# Digital Ulcers and Ventricular Arrhythmias as Red Flags to Predict Replacement Myocardial Fibrosis in Systemic Sclerosis

**DOI:** 10.3390/jcm13010089

**Published:** 2023-12-23

**Authors:** Luna Gargani, Cosimo Bruni, Giancarlo Todiere, Nicola Riccardo Pugliese, Giulia Bandini, Silvia Bellando-Randone, Serena Guiducci, Gennaro D’Angelo, Corrado Campochiaro, Giacomo De Luca, Chiara Stagnaro, Massimo Lombardi, Lorenzo Dagna, Alessia Pepe, Yannick Allanore, Alberto Moggi-Pignone, Marco Matucci-Cerinic

**Affiliations:** 1 Department of Surgical, Medical and Molecular Pathology and Critical Care Medicine, University of Pisa, 56126 Pisa, Italy; 2 Division of Rheumatology, Department of Experimental and Clinical Medicine, Careggi University Hospital, University of Florence, 50121 Florence, Italy; 3 U.O.C. Risonanza Magnetica Specialistica, Fondazione Toscana G. Monasterio, 56124 Pisa, Italy; 4 Department of Clinical and Experimental Medicine, University of Pisa, 56124 Pisa, Italy; 5 Internal Medicine Unit, Department of Clinical and Experimental Medicine, University of Florence, 50121 Florence, Italy; 6 Unit of Immunology, Rheumatology, Allergy and Rare Diseases (UnIRAR), IRCCS San Raffaele Hospital, 20132 Milan, Italy; 7 Department of Rheumatology, Azienda Ospedaliero-Universitaria Pisana, 56126 Pisa, Italy; 8 Multimodality Cardiac Imaging Section, Policlinico San Donato, 20097 Milan, Italy; 9 Institute of Radiology, Department of Medicine, University of Padua, 35122 Padua, Italy; 10 French National Institute of Health and Medical Research (INSERM) U1016, Université de Paris, Hôpital Cochin, 75014 Paris, France

**Keywords:** systemic sclerosis, digital ulcers, cardiac magnetic resonance, late gadolinium enhancement, cardiac involvement, myocardial fibrosis, ECG Holter monitoring

## Abstract

Background: Cardiac involvement in systemic sclerosis (SSc) affects the prognosis of the disease. Echocardiography is the first line imaging tool to detect cardiac involvement, but it is not able to routinely detect myocardial fibrosis. Late gadolinium enhancement (LGE) cardiovascular magnetic resonance (CMR) is the gold standard for replacement myocardial fibrosis assessment, but its availability is currently limited. Aim: We aimed to assess the clinical and instrumental parameters that would be useful for predicting the presence of LGE-CMR, to achieve a better selection of patients with SSc that could benefit from third-level CMR imaging. Methods: 344 SSc patients underwent a comprehensive echocardiogram and LGE-CMR on the same day; for 189 patients, a 24 h ECG Holter monitoring was available. Results: CMR showed non-junctional replacement myocardial fibrosis via LGE in 25.1% patients. A history of digital ulcers (OR 2.188; 95% C.I. 1.069–4.481) and ventricular arrhythmias at ECG Holter monitoring (OR 3.086; 95% C.I. 1.191–7.998) were independent predictors of replacement myocardial fibrosis. Conclusions: CMR can detect patterns of clinical and subclinical cardiac involvement, which are frequent in SSc. A history of digital ulcers and evidence of ventricular arrhythmias at ECG Holter monitoring are red flags for the presence of replacement myocardial fibrosis in CMR. The association between digital ulcers and myocardial fibrosis suggests that a similar pathological substrate of abnormal vascular function may underlie peripheral vascular and cardiac complications.

## 1. Introduction

Systemic sclerosis (SSc) is a complex, clinically heterogeneous disease [1], characterized by extensive vascular alterations, autoimmunity and fibrosis [2]. Cardiac involvement in SSc may affect the endocardium, myocardium and pericardium separately or concomitantly [3], with varying prevalence depending on the definition used [4]. Myocardial fibrosis is the pathological hallmark of myocardial involvement and has been reported in >50% of cases in necropsy studies [5]. Myocardial involvement significantly affects the prognosis of the disease [6], but it is often clinically underestimated [7]; thus, early detection and monitoring are crucial in SSc management [8,9]. Transthoracic echocardiography (TTE) is the routine imaging tool used to assess cardiac involvement, but it is not able to detect myocardial fibrosis, unless more advanced tools such as integrated backscatter and speckle tracking are employed. Comprehensive TTE reports a minority of patients with left ventricular (LV) systolic dysfunction—evaluated by means of ejection fraction (EF)—whereas diastolic dysfunction is more prevalent. When using more sensitive tools, such as Tissue Doppler Imaging (TDI) or strain rate analysis, a higher prevalence of impaired LV and/or right ventricular (RV) contractility has been reported [10,11,12,13]. Contrast imaging based on late gadolinium enhancement (LGE) cardiovascular magnetic resonance (CMR) is currently considered the non-invasive gold standard for imaging replacement myocardial fibrosis in both ischemic and non-ischemic heart disease, and can detect subclinical SSc-related cardiac involvement, which is frequent in SSc [14,15] and has prognostic relevance [16,17,18]. Unfortunately, no clear red flags have been proposed to indicate the presence of early myocardial fibrosis that may help the rheumatologist to refer patients for this advanced imaging investigation. The aim of this study was to assess the clinical and instrumental parameters that may be useful for predicting the presence of myocardial fibrosis in SSc and achieving a better selection of patients that could benefit from third-level CMR imaging.

## 2. Methods

SSc patients, classified according to the ACR/EULAR 2013 criteria [19], were evaluated and consecutively referred to CMR. The inclusion criterion was the absence of absolute contraindications to undergo CMR using a contrast medium. Exclusion criteria were: (1) inability to provide informed consent; (2) age < 18 or > 85 years old; (3) a previous diagnosis of significant left-sided cardiac involvement not related to SSc; (4) a previous diagnosis of significant left-sided cardiac involvement related to SSc; (5) a glomerular filtration rate < 30 mL/min/1.73 m^2^. The local Ethical Committee approved the protocol, and all patients gave written, informed consent. The study was conducted according to the principles of the Declaration of Helsinki.

### 2.1. Clinical Features

At the time of the CMR study, SSc-related clinical information was collected. This was separated into demographics and lifestyle (age, sex, disease duration from the first non-Raynaud’s phenomenon sign or symptom, smoking habits, diabetes mellitus), clinical (including cutaneous subset, modified Rodnan skin score [20], history or presence of digital ulcers (DUs), presence of meaningful dyspnea defined as New York Heart Association functional class > 2, history of chest pain, palpitations or syncope), laboratory (positivity for anti-topoisomerase I or anti-centromere antibody, creatinine clearance, erythrocyte sedimentation rate, NT-proBNP [21]) and pulmonary parenchymal involvement (interstitial lung disease on HRCT, forced vital capacity (FVC%) and diffusion of the lung for carbon oxide (DLCO%)) [22,23] data.

### 2.2. Cardiovascular Magnetic Resonance 

The CMR study was performed on the same day as the TTE with a dedicated 1.5 Tesla (Signa Hdx, General Electrics Healthcare, Milwaukee, WI, USA). An eight-channel cardiac phased array receiver surface coil with breath-holding in end-expiration and ECG-gating were used [13]. 

For the quantification of biventricular function parameters, short axis cine images from the mitral plane valve to the LV apex were acquired using a steady-state free precession (SSFP) pulse sequence with the following parameters: 30 phases, slice thickness, no gap, eight views per segment, FOV 30 cm, phase FOV 1, matrix 224 × 224, reconstruction matrix 256 × 256, a 45 flip angle, TR/TE equal to 3.5/1.5 and a bandwidth of 125 kHz, 1 NEX (breath-holding). Analysis of CMR images was performed using a commercially available software package (Mass Analysis, Leiden, the Netherlands, version 6) in a standard way. Systolic dysfunction was defined as the presence of LV and/or RV EF < 2 SD from the mean values normalized for age and gender [24]. Atrial areas were measured from the four-chamber view projection in the ventricular end-systolic phase. 

To detect the presence of macroscopic myocardial fibrosis, 2D IR fast GRE T1-weighted short-axis and radial images were acquired 8–18 min after macrocyclic gadolinium-based contrast medium intravenous administration at the dose of 0.2 mmol/kg (late gadolinium enhancement technique). The following parameters were used: field of view of 30 mm, slice thickness of 8 mm, no gap between each slice, repetition time of 4.6 ms, echo time of 1.3, flip angle, matrix 224, reconstruction matrix 256, 1 NEX. The appropriate inversion time was set to null the normal myocardium (range 250–200 ms). LGE was considered present when visualized in two different views. For the analysis and correlations with other parameters, we excluded patients with LGE limited to the interventricular junctions (isolated LGE at the right ventricular insertion points) because of low specificity [25]. 

### 2.3. Transthoracic Echocardiography

All patients underwent transthoracic echocardiography examinations with commercially available ultrasound machines (IE33 Philips Medical Systems, Andover, MA, USA) equipped with 2.5–3.5 MHz phased-array probes and second harmonic technology. An ejection fraction was obtained from 2- and 4-chamber views using the biplane disc summation method (modified Simpson’s rule). LV mass was calculated using the Devereux formula and then indexed to body surface area. Tricuspid annular plane systolic excursion (TAPSE) was measured with the M-mode cursor oriented to the junction of the tricuspid valve plane with the right ventricle free wall. The right ventricular–right atrial pressure gradient was derived using the simplified Bernoulli equation from the peak tricuspid regurgitation velocity. Inferior vena cava (IVC) was reported, and a dilated IVC (diameter > 21 mm) that collapsed < 50% with a sniff was considered abnormal. Valvular regurgitation was qualitatively assessed using color-Doppler, and whenever regurgitation was more than mild, it was quantified according to European Association and Cardiovascular Imaging and American Society Recommendations [26,27].

### 2.4. 24-Hour ECG Monitoring

A standard 24-h ECG Holter monitoring was requested according to the clinical indications of the rheumatologist, and data were stored and analyzed. Patients were considered to have ventricular arrhythmias when a Lown’s classification grade ≥ 2 was reported (ventricular premature beats (VPB) > 30/h; multiform VPB; repetitive VPB; early VPB).

### 2.5. Statistical Analysis

Continuous variables are expressed as a mean ± SD. Categorical variables are presented as counts and percentages. Univariate comparisons between patients with and without LGE were made with χ^2^, a 2-sample t-test or a Mann–Whitney U test, as appropriate. The association of selected variables with the presence of LGE was assessed via logistic regression analysis using univariate and stepwise multivariate procedures. Variables were selected according to their clinical relevance and potential impact on cardiac function and myocardial fibrosis. Odds ratios (OR) with the corresponding 95% confidence interval (CI) were estimated. Correlations between parameters were assessed using nonparametric Spearman correlation coefficient analysis or Pearson correlation, as appropriate. A *p*-value < 0.05 was considered statistically significant. All analyses were conducted with the Statistical Package for the Social Sciences (SPSS Inc., Chicago, IL, USA, version 20) and GraphPad Prism (GraphPad Software Inc., San Diego, CA, USA, version 6).

## 3. Results

From the initial population of 344 patients classified according to the ACR/EULAR 2013 classification criteria for SSc [19] and referred to CMR (306 women, mean age 50.3 ± 14.9 years), two patients did not complete the CMR exam (one due to claustrophobia and one due to low compliance). In eight additional patients, CMR was performed but without the LGE technique because of inability to find a peripheral vein into which to inject gadolinium, mainly due to extreme skin thickness. No complications occurred during the CMR, and the gadolinium-based contrast medium did not yield a significant adverse reaction in any patient.

The majority of the patients (50.9%) did not have any symptoms related to cardiac involvement (dyspnea, chest pain, palpitations). Only 19 patients were in NYHA class III or IV; 13 patients reported atypical chest pain and 37 patients had palpitations. 

**Figure 1:**
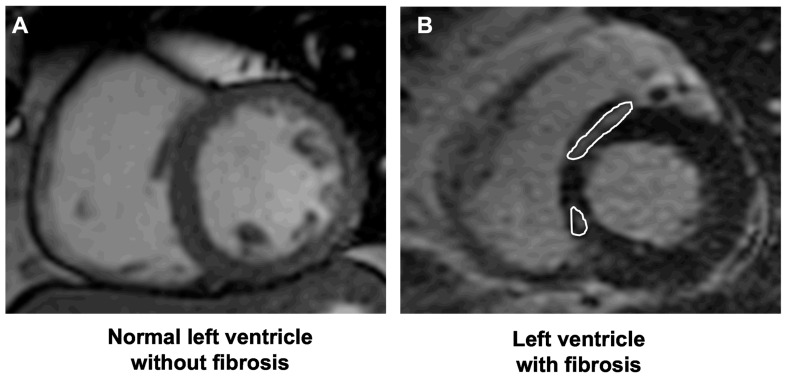
CMR appearance of a normal left ventricle without LGE indicating replacement myocardial fibrosis (**A**) compared to a left ventricle with LGE indicating replacement myocardial fibrosis (**B**) (indicated by the white circles).

The CMR showed replacement myocardial fibrosis (positive LGE with negative T2-weighted images) in 109/334 (32.6%) patients (Figure 1). 

We found that 84/334 (25.1%) of the patients had non-junctional LGE. The majority of patients had a non-ischemic pattern (intramural LGE) involving the interventricular septum (47/84, 56%) and inferolateral wall (31/84, 37%). Three patients had an ischemic distribution pattern of LGE (one in the anterior descending coronary artery territory, one in the right coronary artery territory and one in the circumflex coronary artery territory). Imaging and clinical characteristics of patients classified according to the presence of LGE are shown in Table 1.

Data for ECG Holter monitoring were available in 189 (55%) patients; an association was found between LGE presence and Lown’s classification grade ≥ 2 (χ^2^ = 12.9, *p* < 0.0001). 

Following multivariate analysis, among clinical evaluation and biochemical and functional parameters, only the history of DUs and the presence of ventricular arrhythmias at ECG Holter monitoring (Lown’s classification grade ≥ 2) were independent predictors of the presence of replacement myocardial fibrosis (Table 2). 

**Figure 2:**
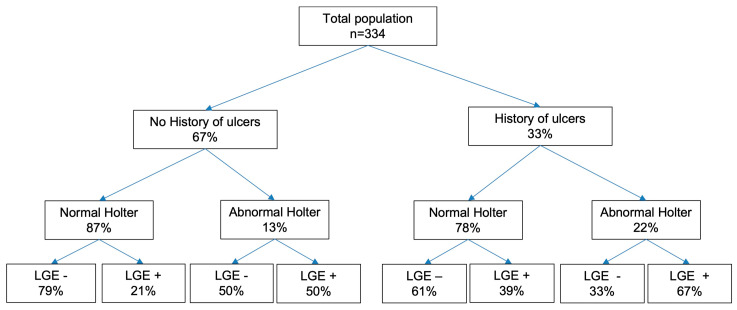
Flowchart stratifying the study population according to history of ulcers, ventricular arrhythmias of ECG Holter monitoring (Lown’s classification grade ≥ 2) and presence of non-junctional LGE.

Specifically, the prediction model combining history of digital ulcers and abnormal Holter ECG monitor had an area under the Receiver Operating Characteristics curve (AUROC) of 0.66 (95% CI 0.57–0.74, *p* < 0.001). The prevalence of ECG Holter abnormalities and a history of DUs in patients with and without CMR-LGE are shown in Figure 2, to support clinical management in referring for CMR.

In patients without DU history and without ventricular abnormalities at ECG Holter, the prevalence of CMR-LGE was 21%, whereas in patients with a history of digital ulcers and ventricular abnormalities at ECG Holter, the prevalence of CMR-LGE was 67%. The prevalence was intermediate in patients without a history of digital ulcers but with the presence of ventricular abnormalities (50%), and in patients with a history of digital ulcers but without ventricular abnormalities (39%). In “false negative” cases (no DU history and no ventricular abnormalities at ECG Holter but the presence of CMR-LGE), the only parameter that could help further classification was a TAPSE ≤ 22 mm, which was present in all but one of these patients.

## 4. Discussion

This is the first large study on SSc assessing the clinical and instrumental determinants of replacement myocardial fibrosis evaluated via CMR. Our data show the frequency and distribution of myocardial fibrosis via LGE, with clinical and instrumental correlations. Consistent with previous studies, we found that a large proportion of SSc patients without a known history of cardiac involvement have a pattern of non-ischemic myocardial fibrosis. Previous studies have underlined that replacement myocardial fibrosis is frequently found via CMR in SSc patients [20,21,22,23,24,25,26,27], mostly at basal and mid-cavity segments of the LV, and often involving the inferolateral septum [20,22,23,26]. Also, the presence of LGE proved to be a valuable prognosticator of major adverse cardiovascular events, independently from the E/e′ ratio in TTE and LVEF or RVEF obtained using CMR [28].

To avoid misinterpretation of the CMR findings and, in particular, of the questionable role of LGE at right ventricular insertion sites, which may be related to expanded extracellular space rather than to replacement fibrosis, we considered in the analysis only those patients with non-junctional localization of LGE. We found that the most robust independent clinical determinants of myocardial fibrosis are the history of digital ulcers and ventricular abnormalities at ECG Holter monitoring.

Microcirculation impairment and related vasculopathy are hallmarks of SSc [28,29] and we might hypothesize that DUs and myocardial fibrosis share the same pathological substrate of remodeling of the vasculature. In SSc, abnormal vascular function may result in DUs in the periphery and cause fibrotic spots in the myocardium when affecting the coronary microcirculation. The damage is, in fact, due to a combination of both repeated ischemia due to vasoconstriction and inflammation. Previous evidence supports the presence of a cardiac Raynaud’s phenomenon, which progressively leads to cardiac tissue damage [30,31]. This is in line with the pathogenetic processes leading from peripheral vasculopathy to the onset of DUs [29]. Additionally, DUs have been demonstrated to be an independent risk factor for a more severe disease course in SSc, including the future development of cardiovascular events [32], stressing the link between peripheral vasculopathy and cardiac involvement.

Our findings are consistent with Allanore et al., who analyzed the large European Scleroderma Trials and Research (EUSTAR) database, finding that prevalence of LV dysfunction in SSc (defined as LVEF < 55% at TTE) is independently associated with age, male gender, myositis, lung involvement and DUs [33]. These data are also in agreement with the evidence that DUs are among the few predictors of worsening disease defined by the progression of organ damage [34]. Mavrogeni et al. also demonstrated that the myocardial perfusion reserve index evaluated via CMR in patients with SSc correlates with DUs, but not with other clinical characteristics [35]. Bruni et al. demonstrated that DUs are present in patients with a very early diagnoses of SSc with internal organ involvement and may represent a sentinel sign for early organ involvement in this population [36]. The typical mid-wall, patchy, non-coronary distribution of LGE that was detected in our patients during CMR is a histological hallmark of replacement myocardial fibrosis with contraction band necrosis unrelated to the epicardial coronary artery distribution. Finding concentric intimal hypertrophy is associated with the fibrinoid necrosis of intramural coronary arteries [37,38]. 

In our population, the majority of patients with ventricular arrhythmias at ECG Holter monitoring showed replacement myocardial fibrosis in CMR. In a previous report from our group, the quantitative amount of LGE was also significantly different between patients with and without ventricular arrhythmias at ECG Holter monitoring [39]. We know that the amount of LGE is an independent predictor of rhythm disturbances [40], which are the most frequent cause of death in patients with SSc. These findings may have relevant clinical implications, underlining that the presence of LV myocardial fibrosis can trigger ventricular arrhythmias. The high percentage of patients with LGE among those who have a positive ECG Holter monitoring confirms the role of the ECG Holter monitoring together with DUs as possible gatekeepers for CMR [40,41]. It should also be reiterated that CMR is a third-level cardiac evaluation with unfortunately still limited availability [42]. On the other hand, TTE is largely available, less expensive and more feasible, but no echo feature can effectively differentiate patients with and without replacement myocardial fibrosis in a standard assessment. This may also be linked to the characteristics of our population, which included also patients with very early diagnosis of SSc. In more advanced phases of the disease, echocardiography usually shows more abnormalities that may help to select patients for CMR. Exciting data are also emerging from TTE strain analysis, which can detect very early myocardial alterations, although the clinical role of this tool is still to be clarified [12,13,34,43].

In our population, as is consistent with previous data, the NT-proBNP values correlated to left and right ventricular function parameters, but not to the presence of replacement myocardial fibrosis [44].

We should acknowledge some limitations of this study. Several patients with an early diagnosis of SSc were included in the study population; on the one hand, this strengthens the value of CMR data, showing myocardial fibrosis even in the very early phases of the disease, when TTE can be still completely normal; on the other, our findings cannot necessarily be extrapolated in very advanced phases of SSc, when performing a CMR can also be challenging for patients who have to lie down flat for at least 30 min, controlling their breathing. Another limitation is that Holter ECG monitoring was not available for all patients, because it was requested by the caring rheumatologist on a clinical basis, which could have introduced a selection bias. We did not distinguish between recurrent and isolated DUs, but chronic DUs are far more common in SSc. Moreover, in our patients, T1 and T2 mapping, which is known to be higher in SSc patients compared to controls, was not studied [45,46]. We have previously shown in 31 SSc patients that either myocardial fibrosis (detected by LGE) and interstitial remodeling of the myocardial and skeletal muscles (detected by an increased extracellular volume fraction) are present, even with normal biventricular function [38]. Having performed this study with standard CMR could be considered a limitation, but it reflects the current standard of care in clinical practice. Obviously, the use of T1 mapping would be more sensitive, but the availability may still be limited. Dumitru et al. have confirmed that patients with SSc have a lower myocardial perfusion reserve in CMR compared to healthy controls, and significantly higher ECV, suggestive of diffuse fibrosis; in these patients, ECV and myocardial perfusion reserve were associated with the presence of digital ulcers at multivariate analysis [47]. Finally, we could not include troponin or other cardiac enzymes in our model, given the high number of missing data we had for this variable. 

In conclusion, standard CMR can detect patterns of replacement myocardial fibrosis as subclinical cardiac involvement, which is frequent in SSc. In CMR, replacement myocardial fibrosis is more frequently linked to a history of DUs and to ventricular arrhythmias. The association between a history of DUs and replacement myocardial fibrosis may suggest a common pathological substrate pointing to an abnormal vascular function underlying cutaneous and cardiac complications, whereas the presence of ventricular arrhythmias can be the undesirable consequence of the disarray induced by collagen deposition in the myocardial interstitial space [48,49]. There is consensus on the added value of CMR in the detection of SSc-related cardiac involvement, even in the early, asymptomatic phases [9]. Given the difficulty of accessing CMR in certain areas, supporting patient selection for this evaluation is an unmet need. In SSc, the combination of these two easily accessible and routinely collected findings may justify the decision-making process of referring patients for third-level imaging such as CMR. Consequently, the presence of DUs and the evidence of ventricular arrhythmias at ECG Holter may be considered useful red flags for the presence of myocardial fibrosis during CMR.

## Figures and Tables

**Table 1:** Different parameters in patients with and without myocardial fibrosis at CMR.

Variable	Negative LGE (n = 250)	Positive LGE (n = 84)	*p*-Value
Female sex (n, %)	224 (90%)	78 (93%)	0.26
Age (years)	49.6 ± 15.1	52.6 ± 14.2	0.10
NYHA class > 2	10 (4%)	9 (11%)	0.025
Atypical chest pain	112 (45)	51 (61%)	0.44
Palpitations	55 (22%)	41 (49%)	0.03
Syncope	3 (1%)	8 (10%)	0.006
Diabetes Mellitus (n, %)	5 (2%)	7 (8%)	0.013
Duration of disease (years)	4.9 ± 6.6	4.8 ± 5.3	0.40
Diffuse cutaneous subset (n, %)	54 (22%)	29 (35%)	0.014
Modified Rodnan skin score	3.5 ± 6.3	3.4 ± 6.5	0.97
Scl-70 antibodies (n, %)	85 (34%)	29 (35%)	0.47
Anti-centromere antibodies (n, %)	86 (34%)	36 (42%)	0.13
History of digital ulcers (n, %)	71 (28%)	38 (45%)	0.002
Current digital ulcer (n, %)	21 (8%)	14 (17%)	0.024
Creatinine clearance (mL/min)	96 ± 27	94 ± 34	0.83
ESR (mm/h)	19 ± 16	22 ± 16	0.15
Abnormal Holter ECG monitor	12 (5%)	22 (31%)	<0.0001
NT-proBNP (ng/L)	195 ± 526	201 ± 257	0.92
ILD on HRCT (n, %)	117 (47%)	46 (55%)	0.22
FVC (%)	111 ± 25	107 ± 28	0.27
DLCO (%)	76 ± 18	68 ± 19	0.001
LV EF (%)	64 ± 5	63 ± 6	0.28
E/e’	8.1 ± 2.6	8.5 ± 2.7	0.40
PASP (mmHg)	25.5 ± 6.3	27.1 ± 8.4	0.07
LA volume index (mL/m^2^)	26 ± 7	27 ± 7	0.26
TDI S’ RV (cm/sec)	13.7 ± 2.6	13.1 ± 2.5	0.15

NYHA = New York Heart Association; ESR = Erythrocyte Sedimentation Rate; NT-proBNP = N-terminal pro–brain natriuretic peptide; FVC = forced vital capacity; DLCO = carbon monoxide diffusing capacity; ILD = interstitial lung disease; HRCT = high resolution computed tomography; LV = left ventricle; EF = ejection fraction; PASP = pulmonary artery systolic pressure; LA = left atrium; TDI = Tissue Doppler Imaging; RV = right ventricle.

**Table 2:** Univariate and multivariate analysis to predict presence of LGE at CMR.

Variable	Univariate Analysis		Multivariate Analysis	
	OR 95% CI	*p*	OR 95% CI	*p*
Age (years)	1.011 (0.997–1.031)	0.108		
NYHA > 2	2.910 (1.139–7.434)	0.026	2.868 (0.673–12.227)	0.154
Diffuse cutaneous subset	1.919 (1.115–3.303)	0.019	0.983 (0.443–2.181)	0.967
Scl-70 positivity	1.057 (0.624–1.790)	0.837		
Anti-centromere positivity	1.432 (0.908–2.259)	0.123		
History of digital ulcers	2.271 (1.350–3.823)	0.002	2.188 (1.069–4.481)	0.032
Diabetes mellitus	4.476 (1.381–14.512)	0.013	0.823 (0.097–7.020)	0.859
NT-proBNP (ng/L)	1.000 (0.909–1.001)	0.936		
ILD on HRCT	1.365 (0.842–2.212)	0.206		
DLCO (%)	0.976 (0.962–0.990)	0.001	0.99 (0.969–1.012)	0.370
PASP (mmHg)	1.033 (0.991–1.077)	0.130		
Abnormal Holter ECG monitor	4.109 (1.836–9.196)	0.001	3.086 (1.191–7.998)	0.020

CI = Confidence Interval; NYHA = New York Heart Association; NT-proBNP = N-terminal pro–brain natriuretic peptide; DLCO = carbon monoxide diffusing capacity; OR = Odds Ratio; PASP = pulmonary artery systolic pressure; ILD = interstitial lung disease; HRCT = high resolution computed tomography.

## Data Availability

The data underlying this article will be shared on reasonable request to the corresponding author. The data are not publicly available due to data protection.
